# Inflammatory Biomarker Profiles in Very Preterm Infants within the Context of Preeclampsia, Chorioamnionitis, and Clinically Diagnosed Postnatal Infection

**DOI:** 10.3390/pediatric15030044

**Published:** 2023-08-10

**Authors:** Jordan T. Ewald, Baiba Steinbrekera, Jennifer R. Bermick, Donna A. Santillan, Tarah T. Colaizy, Mark K. Santillan, Robert D. Roghair

**Affiliations:** 1 Roy J. Carver Department of Biomedical Engineering, University of Iowa, Iowa City, IA 52242, USA; jordan-ewald@uiowa.edu; 2 Department of Pediatrics, University of South Dakota, Sioux Falls, SD 57069, USA; baiba.steinbrekera@sanfordhealth.org; 3 Stead Family Department of Pediatrics, Carver College of Medicine, University of Iowa, Iowa City, IA 52242, USA; jennifer-bermick@uiowa.edu (J.R.B.); tarah-colaizy@uiowa.edu (T.T.C.); 4 Department of Obstetrics and Gynecology, Carver College of Medicine, University of Iowa, Iowa City, IA 52242, USA; donna-santillan@uiowa.edu (D.A.S.); mark-santillan@uiowa.edu (M.K.S.)

**Keywords:** adipokine, chemokine (C-C motif) ligand 2, interleukin-6, leptin, neonatal, preeclampsia

## Abstract

Preterm delivery can be precipitated by preeclampsia or infection, and preterm infants are at heightened risk of postnatal infection. Little is known about the ontogeny of inflammatory biomarkers in extremely preterm infants. We hypothesized that suspected prenatal infection (clinical chorioamnionitis or spontaneous preterm labor) and clinically diagnosed postnatal infection would be associated with unique biomarker signatures, and those patterns would be influenced by the degree of prematurity. Venous blood was collected daily for the first week and weekly for up to 14 additional weeks from 142 neonates born at 22–32 weeks gestation. A custom array was utilized to measure monocyte chemoattractant protein-1 (MCP-1) and interleukin-6 (IL-6). C-reactive protein (CRP) levels were obtained from the electronic medical record. Independent of gestational age, MCP-1 was significantly increased (*p* < 0.001) in association with maternal preeclampsia, but MCP-1 was decreased (*p* < 0.01), and CRP was increased (*p* < 0.01) in the presence of chorioamnionitis with funisitis. IL-6 and CRP were both increased in infants diagnosed with postnatal infection, with peak levels observed on days 2 and 3, respectively. In conclusion, suspected prenatal and postnatal infections and non-infectious complications of pregnancy are associated with unique biomarker profiles, independent of gestational age, including over a 2-fold increase in MCP-1 among newborns of mothers with preeclampsia. Further, in those clinically diagnosed with a postnatal infection in the absence of antenatal infection concerns, IL-6 increases before CRP, emphasizing a potential role for expanded biomarker screening if antibiotics are initially avoided in infants delivered for maternal indications.

## 1. Introduction

Advances in neonatology have increased the survival rates of premature infants, yet long-term complications remain a major consequence of prematurity [1]. Complications in the neonatal period, including respiratory failure, intraventricular hemorrhage, and sepsis, are closely associated with the degree of prematurity [1,2,3]. Longer-term complications, including neurodevelopmental disabilities and social deficits, are, in turn, strongly associated with the prior development of those short-term morbidities [4]. Clinicians are challenged to aggressively treat neonates with potential sepsis to avoid the profound complications that can follow delayed antibiotic initiation [5], but also avoid antibiotic use in infants without clear signs of infection to minimize antibiotic resistance or alterations in the microbiome [6]. However, diagnosis has historically relied on nonspecific blood counts and bacterial cultures that can take days to result. For these reasons, there is a demand for fast and reliable markers of neonatal infection in those where there is a desire to avoid initiating antibiotic therapy immediately upon delivery.

While several biomarkers have been investigated to clarify their roles in improving neonatal care and antibiotic stewardship, several potential markers of inflammation are of particular interest [7]. Interleukin-6 (IL-6) is a pleiotropic cytokine produced during the acute phases of inflammation, and it modulates the inflammatory response of CD4 and CD8 T cells and B cells [8]. C-reactive protein (CRP) is produced primarily in the liver, and its production significantly increases in response to pro-inflammatory cytokines to amplify the activation of the complement system [9]. IL-6 and CRP are often used in concert to aid in the diagnosis of sepsis [10]. Monocyte chemoattractant protein-1 (MCP-1) is a chemokine prominently involved, among other roles, in the recruitment of leukocytes to areas of inflammation [11], and it is prominently altered in histological chorioamnionitis [12]. As such, MCP-1 is thought to play a role in sepsis-induced acute kidney injury, a major contributor to the morbidity and mortality of early-onset neonatal sepsis [13].

Several factors have limited the widespread use of biomarkers in the diagnosis of neonatal sepsis, including assay challenges and a lack of normative values for infants across gestational ages with variable severities of other illnesses. For example, Qiu and colleagues reported cutoff levels of IL-6 for the diagnosis of sepsis in moderately preterm infants in the context of premature rupture of membranes, yet they did not extrapolate to uncomplicated births or other maternal morbidities [14]. While baseline hormone levels have been reported for term and moderately preterm neonates (born at 32 to 34 weeks gestation) [15,16,17], there are insufficient reference data for extremely preterm infants (born before 32 weeks gestation). Filling these gaps in the literature offers the potential for the clinical use of biomarkers to more accurately and appropriately dictate care for this vulnerable population [18].

The aims of this study were to investigate the relationship amongst inflammatory biomarkers in neonates within the first week of life and further report on biomarker levels within an extremely preterm population. We hypothesized that suspected prenatal infection and the diagnosis of postnatal infection would be associated with unique biomarker signatures, and those patterns would be influenced by the degree of prematurity. In addition to providing normative values and clarifying the benefits of expanded cytokine panels, our results reveal distinct biomarker profiles in infants that can inform future mechanistic studies, including significantly increased MCP-1 among infants delivered due to maternal preeclampsia and significantly increased CRP among infants subsequently identified to have chorioamnionitis plus funisitis on placental pathology.

## 2. Materials and Methods

All infants born between 22- and 32-weeks of gestation, without congenital anomalies, admitted to the University of Iowa Stead Family Children’s Hospital neonatal intensive care unit were eligible for enrollment during the 20-month-long recruitment phase [19]. Infants were prospectively enrolled in three predefined birth cohorts, 22 to 25 weeks, 26 to 29 weeks, and 30 to 32 weeks. Informed parental consent was obtained prior to enrollment, and institutional approval was obtained (IRB #201510835). CRP values were retroactively obtained from the electronic medical record (Epic, Verona, WI, USA) as part of the Retrospective Neonatal Research Studies (IRB #201410743). MCP-1 and IL-6 were measured on 200 microliters of blood collected into EDTA tubes daily for 7 days and then weekly until the achievement of either discharge or maturation to 36 weeks postmenstrual age. Samples were obtained during previously scheduled morning lab draws and processed within 4 h with plasma and stored at −80 °C. The plasma samples were analyzed using a customized magnetic bead assay (Millipore Sigma, Burlington, MA, USA) on BioPlex 200 with BioPlex Manager 6.1 software (Bio-Rad, Hercules, CA, USA) [19]. All samples were run in duplicate, alongside 7 standards and a quality control, with our intra-assay correlation coefficient >0.9. All clinically recorded data was stored in REDCap version 8.3.2 (Vanderbilt, Nashville, TN, USA).

The cause of preterm delivery was categorized as suspected infection if there was spontaneous (non-induced) preterm labor, clinically diagnosed chorioamnionitis, or otherwise unexplained fetal distress that prompted preterm delivery. In the absence of those indications, delivery as a consequence of maternal preeclampsia, abnormal placentation (e.g., accreta or previa with abruption), cervical insufficiency, or maternal morbidities (e.g., cancer or heart failure) were categorized as “no suspicion of prenatal infection”. While there can be overlap in the presentation of cervical insufficiency and spontaneous preterm labor, the absence of uterine contractions or labor clinically distinguished the former from the latter. Our institution does not collect universal blood cultures at delivery. In the case of suspected infection, based on the prenatal history and postnatal status, blood cultures may be obtained. When blood cultures are obtained, antibiotics are universally initiated, but there is an automatic stop order placed, and more than 48 h of antibiotics are only administered for persistent concern for infection or culture-proven infection. Given historically low rates of culture-proven sepsis at our institution and a desire to enhance the clinical utility of our investigation by having it conform with our standard of care that completes a full 7 or more day course of antibiotics for infants with a clinical course consistent with infection even if all cultures are negative, postnatal infection was predefined clinically as the administration of 7 or more days of intravenous antibiotics [20].

Statistical analysis was performed using SigmaPlot version 14 (Systat Software, San Jose, CA, USA) and GraphPad Prism version 7.0 (GraphPad Software, La Jolla, CA, USA). Categorical variables were analyzed by Chi-Square tests. Linear regression was utilized to correlate biomarkers with birth weight, chronological age, or postmenstrual age. One-way ANOVA, without correction for multiple comparisons, was utilized to compare biomarker levels across gestational age cohorts, sex, suspected prenatal infection, and diagnosed postnatal infection. Factors that we found significant on univariate analysis (i.e., postnatal age and suspicion for prenatal or postnatal infection) were contrasted by two-way ANOVA with the Holm–Sidak Test for multiple comparisons. Data are presented as mean plus or minus the standard error of the mean, and statistical significance is defined by *p* < 0.05. To minimize the risk of type 1 error, *p* < 0.01 was used to define statistical significance for unplanned subgroup analyses.

## 3. Results

A total of 142 neonates were enrolled in the study across three predefined gestational age cohorts, with 20% born at 22–25 weeks gestation, 36% born at 26–29 weeks gestation, and 44% born at 30–32 weeks (Table 1). The median gestational age at delivery was 29 weeks, the median birth weight was 1.2 kg, and 53% of the infants were male. Infants born at the gestational age of 22 to 25 weeks were more likely than the other cohorts to have suspected prenatal infection (*p* < 0.01) or clinically diagnosed postnatal infection (*p* < 0.01). In contrast, preeclampsia and other maternal morbidities were most common in the cohort born at 30–32 weeks gestation (*p* < 0.01).

Of 142 blood cultures obtained in the first 7 days following delivery (the early-onset sepsis window), only one was positive. In that case, the blood culture from admission grew *Escherichia coli*. Two additional infants had positive blood cultures after day 7 and were diagnosed with late-onset sepsis; one infant had consecutive blood cultures with *Staphylococcus epidermidis*, and the other had a single blood culture with *Klebsiella pneumonia*. For each of the three infants that had positive blood cultures, prenatal infection had been suspected prior to admission based on unexpected preterm labor despite no sign of chorioamnionitis, and postnatal early-onset infection happened to also be diagnosed for all three based on their clinical status. Placental pathology ultimately showed chorioamnionitis and funisitis for the infant with a positive blood culture on admission, but consistent with a presumed non-horizontal transfer of infection for their late-onset sepsis, placental pathology was normal for the two infants with positive blood cultures after day 7.

**Figure 1:**
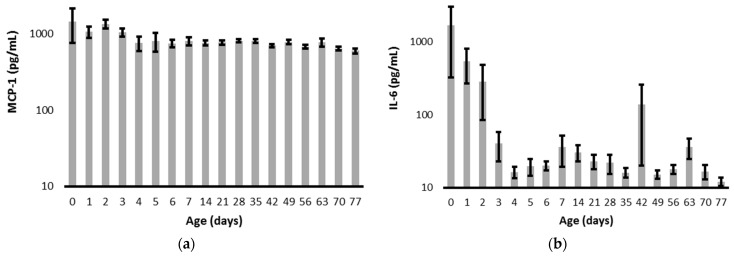
Longitudinal values for (**a**) MCP-1 and (**b**) IL-6. Values are presented as mean plus or minus the standard error of the mean and are presented with a log scale. The IL-6 level on day 42 was influenced by a value of 10,424 pg/mL obtained from an infant that developed an acute exacerbation of chronic multi-organ failure.

Overall, MCP-1 and IL-6 levels declined within 4 days of delivery, and the levels then persisted relatively unchanged through day 77 (Figure 1). The infant with a positive blood culture for *E. coli* on admission had a peak IL-6 of 3887 pg/mL and MCP-1 of only 273 pg/mL on day 1. The CRP on admission was 3.2 mg/dL, and it increased to a peak of 14.7 on day 2. The infant with late-onset *K. pneumonia* sepsis had a protracted course of multi-organ failure with a peak MCP-1 of 3395 pg/mL, IL-6 of 10,424 pg/mL, and CRP of 13.2 mg/dL. The infant with *S. epidermidis* sepsis had MCP-1 levels of 655 ± 115 pg/mL (N = 12), IL-6 levels of 16 ± 3 pg/mL (N = 12), and all five of their CRP levels were <0.5 mg/dL. The low biomarker levels in the presence of positive cultures for *S. epidermidis* are consistent with the findings of others and the relatively low virulence nature of that infection [21].

By linear regression across the first 7 days, chronologic age was significantly correlated with both MCP-1 (R = 0.16, *p* = 0.03) and IL-6 (R = 0.17, *p* = 0.01). The biomarkers did not vary between male and female infants or across gestational age cohorts, although the difference between gestational age cohorts did approach statistical significance for IL-6 (Table 2). By univariate analysis, MCP-1 levels were increased in infants born by Cesarean section, born without preterm labor, and born without suspected prenatal infection, while IL-6 and CRP levels were increased in infants diagnosed with postnatal infection, and CRP was increased in those born after preterm labor (Table 2).

**Figure 2:**
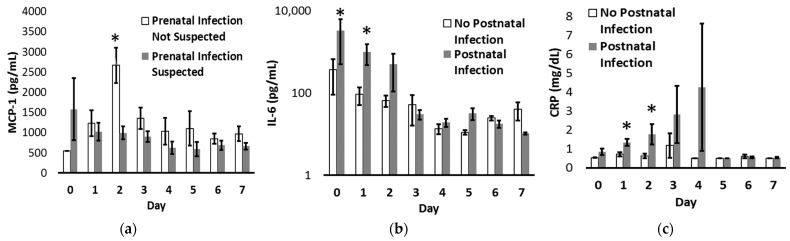
MCP-1, IL-6, and CRP levels from birth to postnatal day 7. (**a**) Compared to neonates born with suspected prenatal infection (gray bars), neonates delivered without suspected prenatal infection (white bars) had increased plasma MCP-1 on day 2. (**b**,**c**) Compared to neonates without postnatal infection (white bars), neonates clinically diagnosed with a postnatal infection (gray bars) had increased plasma IL-6 on days 0 and 1 with increased CRP on days 1 and 2. IL-6 levels are graphed on a log scale. * *p* < 0.05.

Given the sharp decline in cytokine levels over the critical first 7 days after delivery (Figure 1), we further interrogated the temporal association of MCP-1 values with clinically suspected prenatal infection and IL-6 values, as well as CRP levels, with clinically diagnosed postnatal infection (Figure 2). MCP-1 levels were significantly increased on day 2 for those without suspected prenatal infection, and for those diagnosed with postnatal infection, IL-6 levels were significantly increased on postnatal days 0 and 1, while CRP levels were significantly increased on days 1 and 2.

**Figure 3:**
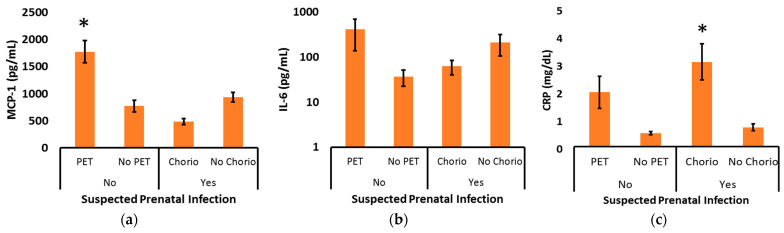
Across the first 7 days after delivery, (**a**) MCP-1, (**b**) IL-6, and (**c**) CRP are contrasted for pregnancies with or without preeclampsia (PET) or clinical chorioamnionitis (Chorio). (**a**) Postnatal MCP-1 levels were increased in pregnancies complicated by PET, and (**c**) postnatal CRP levels were increased in pregnancies complicated by Chorio. * *p* < 0.001 versus no PET or no Chorio.

To further explore the relationship between suspected prenatal infection and the biomarker levels across the first 7 days after delivery, unplanned subgroup analyses were performed by subcategorizing those without suspected prenatal infection based on their PET status and subcategorizing those with suspected prenatal infection based on their clinical chorioamnionitis status (Figure 3). In the absence of suspected prenatal infection, the increase in MCP-1 was strongly associated with the diagnosis of maternal preeclampsia (*p* < 0.001). Likewise, CRP tended to be high in the presence of maternal preeclampsia, but that did not reach statistical significance (*p* = 0.06). In the presence of suspected prenatal infection, a decrease in MCP-1 was seen in those diagnosed with clinical chorioamnionitis, but that did not reach statistical significance (*p* = 0.09). In sharp contrast, postnatal CRP levels were significantly increased in the context of maternal clinical chorioamnionitis (*p* < 0.001).

**Figure 4:**
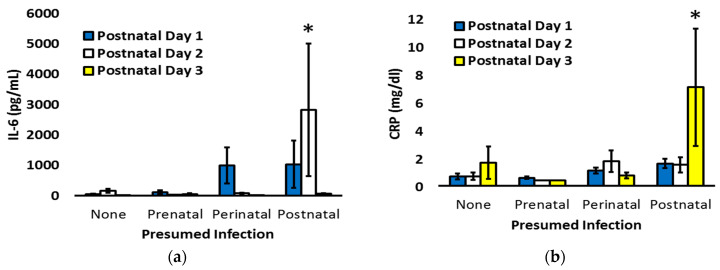
IL-6 (**a**) and CRP (**b**) on postnatal day 1 (blue bars), day 2 (white bars), and day 3 (yellow bars) for infants categorized as having no concern for infection, only prenatal infection, both prenatal and postnatal (perinatal) infection, or only postnatal infection. * *p* < 0.01 versus the other cohorts.

To explore the temporal patterns of CRP and IL-6, the markers significantly associated with postnatal infection, infants were re-classified as having no concern for infection, only suspected prenatal infection, suspected prenatal and postnatal infection (perinatal infection), or only postnatal infection. Two-way ANOVA, with postnatal age and infection status as factors and planned contrast of biomarker levels based on infection status during each postnatal day, was utilized to assess the patterns on days 1 to 3, when clinicians are most often debating the need to initiate or discontinue antibiotic therapy in the context of prenatal and postnatal clinical status (Figure 4). Overall, there was not a statistically significant interaction between postnatal age and infection status for IL-6 (*p* = 0.08), but a significant interaction was present for CRP (*p* < 0.001). Compared to the other cohorts, infants with isolated postnatal infection had increased IL-6 on day 2 (*p* < 0.001) and increased CRP on day 3 (*p* < 0.01).

Although our primary analysis was based on real-time clinical decisions regarding the likelihood of infection, we performed a secondary analysis of biomarker levels based on final placental pathology reports. Of 142 infants, 112 (79%) had placental pathology, 51 (46%) of those placentas had histopathologic chorioamnionitis, and 23 (45%) of the placentas with chorioamnionitis also had funisitis. Histopathologic chorioamnionitis was seen on the pathology for all infants that had previously been clinically diagnosed with chorioamnionitis. By univariate analysis, MCP-1 was decreased (*p* < 0.01) and CRP was increased (*p* < 0.05) in infants with signs of chorioamnionitis on pathology (Table 3). The increase in CRP was specifically associated with the presence of funisitis in addition to histopathologic chorioamnionitis (*p* < 0.01). Only one mother with preeclampsia also had chorioamnionitis, and the chorioamnionitis was only diagnosed retrospectively on placental pathology, and it was not associated with funisitis. That infant had the second-highest MCP-1 levels within the chorioamnionitis-positive cohort (mean of 2078 mg/dL) as well as elevated IL-6 and CRP values on day 1 (2946 pg/mL and 5.2 mg/dL, respectively).

## 4. Discussion

Premature infants are at heightened risk of neonatal complications, and this has contributed to ongoing demand for new diagnostic methods specific to this early phase of life [18]. To help address this, the aim of our study was to analyze inflammation biomarkers levels across gestational ages with a focus on the correlations of IL-6 and MCP-1 with clinically diagnosed infections. While we hypothesized that the degree of prematurity would influence the biomarker levels, univariate analysis of the GA cohorts did not show significance for IL-6, MCP-1, or CRP. This varies from some other hormones, including the adipokine leptin that is produced by specific tissues and affected if tissue development is not complete at the time of delivery [19]. Instead of differences based on the degree of prematurity, the chronological age of neonates had a significant inverse relationship with IL-6 and MCP-1 levels over the first week after delivery. In the process of defining the potential role of these markers in the clinical diagnosis of infection among infants born as early as 22 weeks gestation, our investigations extend the existing literature by contextualizing biomarker levels based on chronological age, maternal preeclampsia, and the clinical suspicion of infection.

We observed a sharp decrease in MCP-1 within days of delivery, and that temporal pattern is consistent with the results seen in 30-week to 32-week gestation infants by Lusyati and colleagues [16]. In our study, infants that were born following pregnancies complicated by clinically suspected infection or histopathologically proven chorioamnionitis had the lowest MCP-1 levels. This contrasts with previous reports showing elevation of MCP-1 in older pediatric patients with sepsis [22], but it is consistent with the chorioamnionitis-induced immune hypo-responsiveness that has been described in several studies [12,23,24]. The etiology of the hypo-responsive transcriptional phenotype of perinatal monocytes has not been fully elucidated [24], but ovine studies suggest the development of endotoxin cross-tolerance following repeated exposure to intra-amniotic cytokines, as elaborated during the evolution of chorioamnionitis [25]. Beyond a potential for infection-related suppression in MCP-1 production, the decreased level of MCP-1 in pregnancies with suspected infection was linked with the increase in MCP-1 levels among infants born to mothers with preeclampsia, our most common cause for preterm delivery in the absence of preterm labor.

Investigations have identified increased maternal MCP-1 levels in the presence of preeclampsia [26,27], but ours is the first investigation we are aware of that has identified increased MCP-1 levels in the offspring of mothers with preeclampsia. MCP-1 is overexpressed in preeclamptic placentas [28], and while it is also known that MCP-1 levels quickly fall in the newborn’s circulation [16], it is not known whether the placenta is a source of neonatal MCP-1. Alternatively, a common humoral or genetic factor might drive increased MCP-1 expression in both mother and offspring. For example, heritable MCP-1 gene variants are associated with increased MCP-1 expression and preeclampsia [29,30], and that could explain some of the increased risk of cardiovascular disease and preeclampsia in the offspring of preeclamptic mothers [31,32]. The increase in MCP-1 among offspring born by Cesarean and without preterm labor is consistent with the delivery of many infants by Cesarean without labor in the presence of preeclampsia, but it is possible other unknown mechanisms contribute to a reduction in MCP-1 following labor and/or vaginal delivery. Ultimately, for the purposes of our investigations, the association of MCP-1 levels with both infectious and non-infectious complications of pregnancy suggests poor specificity for its use in the clinical diagnosis of infection [33].

It is notable that although the increase in CRP levels we observed in infants born to mothers with preeclampsia did not reach statistical significance (Figure 3c, *p* = 0.06), the directionality of the observation paralleled the significant increase in MCP-1 associated with preeclampsia (Figure 3a, *p* < 0.001). This could again reflect a common etiology, such as an increased inflammatory process. In that regard, multiple investigations have demonstrated increased oxidative stress during preeclamptic pregnancies, and there is also the potential for ischemia-reperfusion-related changes after delivery has occurred [34]. With the notable exception of pregnancies complicated by chorioamnionitis, the CRP levels we obtained generally paralleled MCP-1 levels. The marked increase in CRP following the diagnosis of histopathologic chorioamnionitis with funisitis is consistent with an extensive body of literature and likely reflects the intense inflammation that contributes to the features that are hallmarks of chorioamnionitis [35]. Beyond chorioamnionitis, prenatal infection was not associated with an increase in biomarker levels, but that is perhaps not surprising given that over half of the patients with suspected prenatal infection had preterm labor as the index of suspicion, and preterm labor is clearly a multifactorial process. Regarding antenatal infection surveillance and confirmation, based on our results, CRP has value in support of the clinical diagnosis of chorioamnionitis.

Both CRP and IL-6 are established biomarkers for postnatal infection, but their relative utility and specificity are debated [36]. In our investigation, early postnatal CRP levels were increased in association with not just postnatal infection but also with chorioamnionitis. In those with only postnatal infection, the increase in CRP was not observed until day 3. In contrast, IL-6 was significantly increased on the day of birth in the presence of suspected postnatal infection, and marked elevations were seen on day 2 among infants with isolated concern for postnatal infection. The increase in IL-6 prior to the elevation of CRP follows previous literature on the diagnostic capability of IL-6 and CRP together, as reported by Tessema and others [37,38,39], it is also consistent with the well-described effect of IL-6 on subsequent CRP production [40], and it is very reminiscent of the results seen in late-onset culture-proven sepsis [41].

Our study does have some limitations. Our population consisted of 142 neonates from a single institution. While the diagnosis of clinical sepsis might vary across providers and institutions, our study was designed to reflect upon and inform the current management of infection at an institution with historically high rates of periviable infant survival. Because CRP values were obtained retrospectively, there were more values available for infants undergoing higher scrutiny for possible infection, but this again reflects current clinical practice. We do not routinely screen for viral infections beyond the meningitis panel included in cerebrospinal fluid testing, but the aerobic blood cultures that were obtained do detect a wide variety of bacteria and fungi, and all the positive results are reported.

## 5. Conclusions

To our knowledge, we are the first group to report on these three biomarkers and their associations with maternal preeclampsia, chorioamnionitis, and postnatal infections among a cohort of very preterm infants. Independent of gestational age at delivery, our data suggest a role for expanded biomarker screening, including early postnatal IL-6 levels, to potentially allow earlier and more reliable detection of sepsis to promote proper antibiotic use and earlier discontinuation of antibiotics when they are not needed. Future investigations are needed to improve our understanding of the relationship of preeclampsia and chorioamnionitis with postnatal MCP-1 levels. Longitudinal MCP-1 levels and genetic testing could clarify potential transgenerational inheritance of preeclampsia risk, while the association of MCP-1 with both preeclampsia and chorioamnionitis further supports a role for MCP-1 in both normal placentation and acute placental inflammation. The demonstration of increased CRP, specifically among infants with chorioamnionitis plus funisitis, underscores the importance of cataloging surrogate markers of perinatal inflammation that could signal the need for enhanced surveillance for potential adverse outcomes. It is paramount to further investigate the longitudinal biomarker levels of neonates and the impact of clinical interpretation and utilization of those levels on prospective short-term and long-term clinical outcomes of very preterm infants. Ultimately, clinical research bolstered by basic science investigations has the potential to strengthen the associations and enhance our mechanistic understanding.

## Figures and Tables

**Table 1:** Maternal and infant characteristics were obtained for the three predefined cohorts based on gestational age at delivery (22–25, 26–29, or 30–32 completed weeks). Continuous variables are expressed as medians (interquartile range), and categorical variables are expressed as counts. In some cases, prenatal infection was suspected based on the presence of more than one diagnosis.

	All Infants N = 142	22–25 Weeks N = 29	26–29 Weeks N = 51	30–32 Weeks N = 62	*p*
Gestational Age (weeks)	29.4 (26.6, 31.3)	24.3 (23.2, 25.1)	28.0 (27.1, 29.1)	31.8 (31.0, 32.0)	<0.01
Birth Weight (g)	1186 (883, 1610)	624 (554, 757)	1075 (966, 1223)	1655 (1370, 1914)	<0.01
Male Sex	75 (53%)	17 (59%)	22 (43%)	36 (58%)	0.22
Prenatal Infection Suspected	90 (63%)	27 (93%)	33 (65%)	30 (48%)	<0.01
Preterm Labor	83 (53%)	27 (93%)	30 (59%)	26 (42%)	<0.01
Clinical Chorioamnionitis	9 (6%)	4 (14%)	3 (6%)	2 (3%)	0.15
Fetal Distress	7 (5%)	0 (0%)	3 (6%)	4 (6%)	0.39
Prenatal Infection Not Suspected	52 (37%)	2 (7%)	18 (35%)	32 (52%)	<0.01
Preeclampsia	29 (20%)	1 (3%)	9 (18%)	19 (31%)	<0.01
Abnormal Placenta	12 (8%)	0 (0%)	5 (10%)	7 (11%)	0.18
Cervical Insufficiency	4 (3%)	1 (3%)	1 (2%)	2 (3%)	0.90
Maternal Morbidity	7 (5%)	0 (0%)	3 (6%)	4 (6%)	0.39
Postnatal Infection Diagnosed	63 (44%)	21 (72%)	26 (51%)	16 (26%)	<0.01

**Table 2:** Categorical values are presented as mean ± SEM for MCP-1, IL-6, and CRP.

		MCP-1 (pg/mL)	IL-6 (pg/mL)	CRP (mg/dL)
Sex	Female	938 ± 99	213 ± 123	1.1 ± 0.2
	Male	1159 ± 112 *p* = 0.16	331 ± 151 *p* = 0.57	1.0 ± 0.1 *p* = 0.73
Mode of Delivery	Vaginal	821 ± 92	206 ± 149	1.3 ± 0.3
	Cesarean	1189 ± 105 *p* = 0.02	320 ± 134 *p* = 0.60	0.9 ± 0.1 *p* = 0.11
Preterm Labor	Yes	792 ± 74	363 ± 165	1.2 ± 0.2
	No	1487 ± 147 *p* = 0.000007	157 ± 49 *p* = 0.32	0.7 ± 0.1 *p* = 0.04
Gestational Cohort	22–25 weeks	809 ± 186	786 ± 457	1.1 ± 0.2
	26–29 weeks	1180 ± 149	157 ± 64	0.8 ± 0.1
	30–32 weeks	1080 ± 93 *p* = 0.24	174 ± 111 *p* = 0.06	0.7 ± 0.3 *p* = 0.46
Prenatal Infection	Suspected	928 ± 88	288 ± 126	0.8 ± 0.1
	Not Suspected	1388 ± 146 *p* = 0.006	268 ± 170 *p* = 0.93	1.3 ± 0.3 *p* = 0.05
Postnatal Infection	Diagnosed	1175 ± 140	518 ± 213	1.4 ± 0.2
	Not Diagnosed	969 ± 75 *p* = 0.18	71 ± 19 *p* = 0.03	0.7 ± 0.1 *p* = 0.001

**Table 3:** Biomarker levels based on the presence or absence of histopathologic chorioamnionitis (with or without funisitis) on placental pathology are presented as mean ± SEM.

Histopathologic Chorioamnionitis	MCP-1 (pg/mL)	IL-6 (pg/mL)	CRP (mg/dL)
Present (N = 51) With Funisitis (N = 23) No Funisitis (N = 28)	796 ± 91 ** 799 ± 137 ** 792 ± 115 **	427 ± 201 451 ± 269 398 ± 301	1.32 ± 0.23 * 1.83 ± 0.47 ** 0.93 ± 0.16
Not Present (N = 61)	1406 ± 141	104 ± 27	0.82 ± 0.10

* *p* < 0.05 or ** *p* < 0.01 versus Not Present.

## Data Availability

The data presented in this study are available on request from the corresponding author. The data are not publicly available to protect patient confidentiality.
